# Factors influencing the higher incidence of tuberculosis among migrants and ethnic minorities in the UK

**DOI:** 10.12688/f1000research.14476.2

**Published:** 2018-08-20

**Authors:** Sally Hayward, Rosalind M. Harding, Helen McShane, Rachel Tanner

**Affiliations:** 1 St John’s College, University of Oxford, Oxford, OX1 3JP, UK; 2 Department of Zoology, University of Oxford, Oxford, OX2 6GG, UK; 3 The Jenner Institute, University of Oxford, Oxford, OX1 3PS, UK

**Keywords:** Tuberculosis, UK, Migrants, Ethnic minorities, Socio-economic inequality, Stigma

## Abstract

Migrants and ethnic minorities in the UK have higher rates of tuberculosis (TB) compared with the general population. Historically, much of the disparity in incidence between UK-born and migrant populations has been attributed to differential pathogen exposure, due to migration from high-incidence regions and the transnational connections maintained with TB endemic countries of birth or ethnic origin. However, focusing solely on exposure fails to address the relatively high rates of progression to active disease observed in some populations of latently infected individuals. A range of factors that disproportionately affect migrants and ethnic minorities, including genetic susceptibility, vitamin D deficiency and co-morbidities such as diabetes mellitus and HIV, also increase vulnerability to infection with *Mycobacterium tuberculosis (M.tb)* or reactivation of latent infection. Furthermore, ethnic socio-economic disparities and the experience of migration itself may contribute to differences in TB incidence, as well as cultural and structural barriers to accessing healthcare. In this review, we discuss both biological and anthropological influences relating to risk of pathogen exposure, vulnerability to infection or development of active disease, and access to treatment for migrant and ethnic minorities in the UK.

## Introduction

Tuberculosis (TB) is a bacterial disease caused by *Mycobacterium tuberculosis* ( *M.tb*), which most commonly affects the lungs ^1^. *M.tb* infection is acquired by inhalation of infectious particles released from close contacts ^2^. While 10% of those infected develop active disease, the majority of individuals mount an effective immune response leading to successful containment of *M.tb* growth; a condition known as latent *M.tb* infection or LTBI ^3^. Active TB disease results in systemic symptoms including fever, weakness and weight loss, as well as site-specific symptoms. For active pulmonary TB disease, the most common symptoms are persistent coughing (sometimes producing blood) and chest pain ^3^. In 15–20% of active cases, and usually in those with immunosuppression, the infection spreads outside the lungs causing extrapulmonary TB. The symptoms of extrapulmonary TB vary considerably depending on the site of infection, which may include lymph nodes, pleura and osteoarticular areas ^4^. Untreated, the 10-year case fatality rate of active disease is between 54 and 86% in HIV-negative individuals ^5^. LTBI, which is asymptomatic, ordinarily has a 5–10% lifetime risk of reactivation ^2^. LTBI may be diagnosed through cutaneous tuberculin skin test (TST) or interferon-γ release assays (IGRA), while clinically suspected TB disease is evaluated through chest radiograph and diagnostic microbiology for acid-fast bacilli. Effective antibiotic treatment is available, but involves long and complex regimens. Furthermore, rates of multi-drug-resistant TB (MDR-TB) and extensively-drug-resistant TB (XDR-TB) are increasing ^6^.

Globalisation, conflict and financial reasons have become increasingly important drivers of migration flows, leading to more permanent migrants moving from low/middle income to high-income countries ^7^. Studies of the incidence of TB in migrant communities in several high-income countries including Western Europe, the United States and Australia provide important evidence of disparity between subpopulations ^8– 12^. In the UK, a substantial proportion of foreign-born migrants arrive from former colonies in sub-Saharan Africa and the Indian Subcontinent (ISC) ^13^. Incidence of TB disease is higher among all migrant and ethnic minority groups living in the UK compared with the UK-born population. In 2016, 73.6% of individuals with diagnosed TB disease were foreign-born, with India and Pakistan the most frequent countries of birth among such cases ^14^. While TB rates have been falling slowly across all UK populations since 2011, they remain 15 times higher in the foreign-born than the UK-born population. Furthermore, within the UK-born population, non-white ethnic groups had TB rates 3 to 14 times higher than the white ethnic group ^14^. There is much heterogeneity in both absolute number of cases and incidence rates (per 100,000 of population group) among migrants from different countries and among different ethnic groups. While number of cases is confounded by size of population group, variation in incidence rate reflects varying levels of risk for different migrant and ethnic groups. Migrants from the ISC (India, Pakistan and Bangladesh) and black ethnic groups demonstrate particularly high incidence ^15^.

In explaining this disparity, the media have claimed that migrants and ethnic minorities ‘import’ TB into the UK. For example, according to the *Daily Express*, a TB outbreak in Leicester in 2001 indicated that “we are still vulnerable to infection from other countries” because “immigrants and visitors from less medically-advanced countries can carry the infection into Britain” ^16^. In this way, migrants and ethnic minorities are labelled as carriers of infectious agents ^17^. This popular view attributes the disparity in TB rates solely to differential pathogen exposure. Although exposure to *M.tb* in country of origin plays an important role, such an attitude obscures the mix of factors that increase the vulnerability of migrants and ethnic minorities to development of active TB disease from latent infection, and the influence of differential treatment-seeking behaviour in promoting the spread and therefore incidence of TB within these communities. As King highlights, TB is a complex disease and “the disproportionate burden of TB on certain populations is seldom the result of a single obvious cause” ^18^. In particular, it is difficult to disentangle socio-economic disadvantage from migrant/ethnic cultural identity in analyses of how reactivation of latent infection as well as transmission contribute towards TB incidence in migrant and ethnic minority populations; especially given that their relative importance differs between and within these groups. Nevertheless, it is clear that each factor - from the biological to the social ^19^ - has a role to play and should be considered in strategies to tackle this inequality.

**Figure 1.**
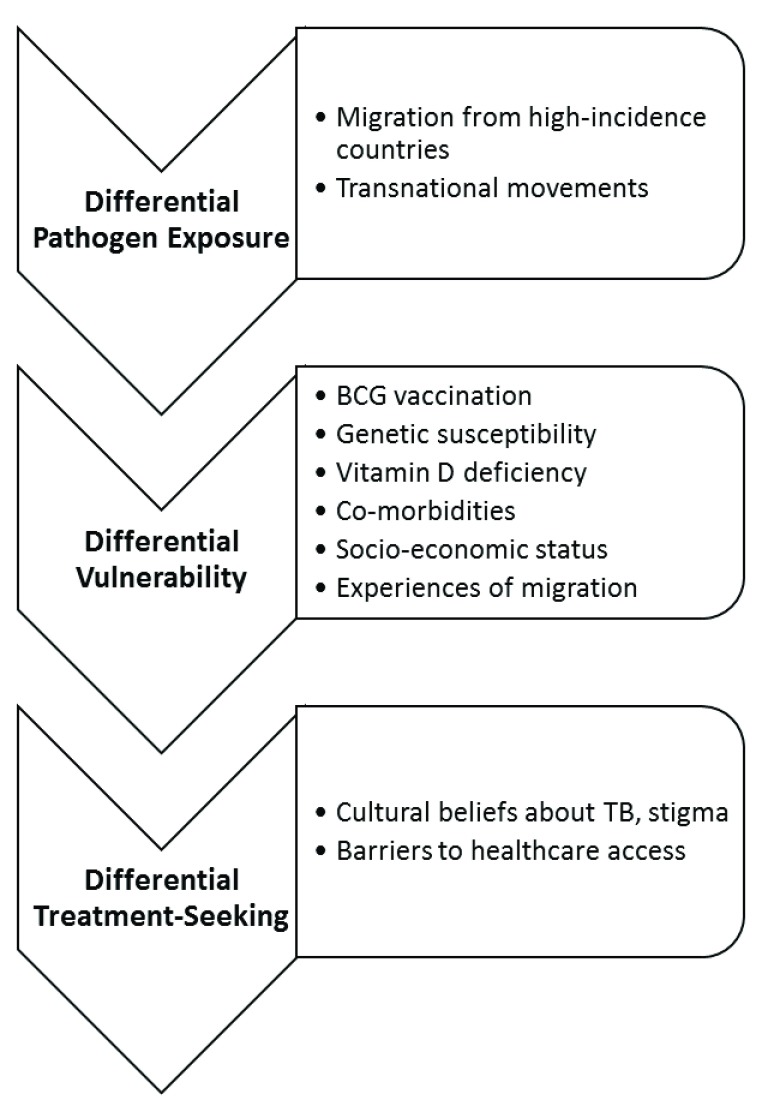
Summary of factors contributing to the increased incidence of TB in migrant and ethnic minorities in the UK.

We discuss the biological, social and cultural factors relating to risk of pathogen exposure, vulnerability to infection or development of active disease, and access to treatment which contribute to the increased incidence of TB in migrant and ethnic minorities in the UK ( Figure 1).

## Epidemiology

The higher burden of TB observed among foreign-born individuals in the UK could be due to arrival of migrants with active TB, reactivation of remotely-acquired LTBI post-arrival, or local transmission ^20^. Meta-analyses of screens for active TB at entry have indicated that only a small proportion (~0.35%) of immigrants have active TB at time of arrival in the EU/EEA ^21, 22^. Since 2012, the UK Home Office has required pre-arrival screening for active pulmonary TB disease for all long-term visa applicants from endemic countries; those diagnosed with active disease are denied a medical clearance certificate ^23^. Thus arrival of migrants with active TB is not thought to contribute significantly to the overall burden of disease among foreign-born individuals in the UK; rather several studies suggest a more prominent role for the reactivation of remotely-acquired LTBI post-arrival ^20, 24, 25^. In the initial years following arrival in a lower incidence setting, migrants with LTBI have a higher risk of reactivation than the host population ^8, 26, 27^.

Local transmission within immigrant communities in the UK may also contribute to the higher incidence of TB cases observed in migrants and ethnic minorities. Such groups are more likely to live in densely-populated areas with a high concentration of their ethnic community, which may foster spread of TB, particularly given the mode of transmission ^28^. Moreover, Bakhshi suggests that larger household size among migrants and ethnic minorities - perhaps due to cultural factors favouring a multi-generational rather than nuclear family structure - increases *M.tb* transmission, as approximately one-third of household contacts will become infected in a household with an active TB case ^29^.

In order to establish the relative importance of local transmission versus reactivation of LTBI, molecular fingerprinting and other strain typing have been used since the 1990s. Genetic clusters have been assumed to represent epidemiologically linked chains of recent transmission, whereas unique isolates have been taken to represent reactivational disease ^30^. Early findings were conflicting, likely due to the inability of such techniques to reliably distinguish past and recent transmission ^31^. The advent of whole-genome sequencing of *M.tb* offered additional resolution, and in one such study of Oxfordshire TB cases in 2007–2012, those born in a high-incidence country were less likely to be part of a recent transmission cluster than those born in a low-incidence country (especially the UK), even when adjusting for social risk factors ^32^. Similarly, Aldridge *et al*. identified only 35 of over 300,000 migrants screened prior to entry into England, Wales and Northern Ireland as assumed index cases for transmission clusters ^33^. In addition, a Canadian study found no evidence to support concerns about transmission from immigrants born in high-incidence countries. These findings suggest that reactivation of LTBI is more important in explaining the higher incidence of TB among migrants than exogenous infection due to local transmission.

## Differential exposure

Rates of active TB disease diagnosed after arrival in the UK correlate with TB incidence in the country of origin ^34^, indicating that differential exposure among migrants is a key factor influencing TB incidence in foreign-born populations. 13% of foreign nationals in the UK are from a country where TB incidence is ≥250 cases per 100,000 ^13^. After the Second World War, substantial numbers of migrants arrived from the Commonwealth and former British Empire; particularly from the ISC. These movements were driven by factors such as Britain’s labour shortages for post-war reconstruction and political turbulence after decolonisation, for example following the creation of Pakistan ^35^. Many Commonwealth countries have high TB incidences: the highest incidences globally are found in Africa (275 per 100,000 population in 2015) and South East Asia (246 per 100,000) ^6^. Migrants from these countries are at a greater risk of having been exposed to *M.tb* and contracting LTBI. Indeed, of the 11 countries that were each the source of more than 2% of foreign-born cases in 2001–2003 (collectively accounting for 73% of foreign-born cases), all were in South Asia or sub-Saharan Africa ^36^.

It is useful to consider migration to the UK from the perspective of transnationalism, defined as “the process by which immigrants forge and sustain multi-stranded social relations that link together their societies of origin and settlement” ^37^. From the 1920s until recently, migration research has tended to focus on the incorporation of migrants in their destination country rather than continued ties with their country of origin. However, since the 1990s, “the transnational turn” has provided “a new analytic optic” ^38^. From this perspective, given that migrants maintain ties across the borders of nation-states, return visits to their country of origin and overseas visitors to the UK may result in increased exposure to *M.tb*. Where data was available, 17.2% of TB cases in 2016 in England had travelled outside the UK (excluding Western Europe, US, Canada, New Zealand and Australia) in the two years before diagnosis, and 7.3% had received an overseas visitor. 23.3% of non-UK born cases had travelled outside the UK in 2016 compared with only 4.7% of UK born cases ^14^. Such movements are increasing as globalisation leads to the intensification of international interconnectedness: in the UK, travel to visit family and friends abroad increased by 67% between 1998 and 2007 ^39^. In 2007, UK residents made nearly 900,000 trips to the ISC for the purpose of visiting friends and family ^39^.

There is some evidence to suggest that travel to countries with high TB incidence increases the risk of acquiring LTBI, with greater risk associated with more prolonged travel and higher TB burden in the destination country ^40^. Such individuals are then at risk of developing active disease after returning to the UK. A study in Blackburn, Hyndburn and Ribble Valley found that 12.8% of active cases among Indian, Pakistani and Bangladeshi ethnic groups occurred within 3 years of revisiting the ISC ^41^. Furthermore, a case-control study in Liverpool found that TB cases were 7.4 times more likely to have recently received visitors from abroad ^42^. A case-control study of patients of ISC ethnic origin in North West England found a weak association between revisiting the ISC and TB cases within the following 3 years ^43^. However, there is currently limited data in this area and further research is needed to determine the proportion of TB acquired through travel ^20^. In particular, there is a need to elucidate whether certain types of travel are associated with a greater risk of acquiring LTBI, for example health professionals or volunteers ^44^.

Second generation immigrants (born in the UK but with parents born overseas) are at lower risk of TB than first generation immigrants (born overseas and migrated to the UK) but still remain at increased risk compared with the general population ^15^. This suggests a role for exposure to *M.tb* in the country of origin, but the higher incidence of TB among ethnic minorities compared with the general population is likely to be partially attributable to transnational connections, transmission within migrant populations, and other factors discussed in this review. However, quantification of the TB burden in this group is challenging as second generation migration is insufficiently captured by the prevailing European surveillance variables, and prospective studies on the risk of TB in successive migrant generations are lacking ^45^.

A further dimension of differential exposure is variation in pathogen factors such as global genomic diversity in the *M.tb* complex. There are six major genetic lineages of *M.tb* found in distinct geographical regions ^46^. Lineages can induce different host immune responses ^47– 49^, and it is possible that *M.tb* strain may contribute to variation in clinical TB phenotype between ethnic groups, for example the higher prevalence of extrapulmonary disease among non-white ethnic than UK-born cases ^50^. However, a study in an ethnically heterogeneous UK population found that ethnicity was strongly associated with clinical TB phenotype independent of *M.tb* lineage, suggesting that host factors may be more important in determining the clinical manifestation of TB ^51^.

## BCG vaccination

*Mycobacterium bovis* Bacille Calmette-Guerin (BCG) is the only currently available vaccine against TB. BCG confers reliable protection against disseminated forms of TB such as miliary disease and meningitis in infants ^52, 53^. However, protection against pulmonary TB (the most common form of disease) varies considerably by geographical region ^54^. While the UK has one of the highest levels of BCG efficacy (~80%), a low level, or complete lack of, protection has been reported in many migrant countries of origin such as India ^54– 56^. The problem may be further confounded by limited access to vaccines and other healthcare in low-income country settings, but prevalence of *M.tb* infection in endemic countries remains high even where there is good BCG coverage ^57, 58^.

It has been hypothesised that exposure to non-tuberculous environmental mycobacteria (NTM), which increases with proximity to the equator, plays a central role in limiting BCG efficacy ^59^. Individuals may develop an immune response to NTM that either ‘masks’ or ‘blocks’ the ability of BCG to induce a protective response ^59– 62^. In a trial in Chingleput, India, 95% of individuals were PPD positive by 15–20 years of age ^63^. In a trial in Malawi where there is high NTM exposure and poor BCG efficacy, individuals with lower immune responses to NTM showed greater IFN-γ responses to BCG ^64^. Furthermore, in mice sensitised with NTMs, the protective effect of BCG (but not a TB subunit vaccine) was considerably reduced ^65^. The low levels of BCG protection found in countries with high TB incidence likely contribute to the prevalence of LTBI among migrant populations.

## Genetic susceptibility

The idea of a heritable component to TB was suggested as early as 1886 by Hirsch: “That phthisis [TB] propagates itself in many families from generation to generation is so much a matter of daily experience, that the severest sceptic [sic] can hardly venture to deny a hereditary element in the case” ^66^. There are several lines of evidence suggesting that host genetic factors may contribute to TB susceptibility and resistance ^67^. Early studies demonstrated that monozygotic twins have a higher risk of developing active TB compared with dizygotic twins ^68, 69^, and several relevant loci have since been identified using candidate gene studies and genome-wide association studies ^70– 75^. Variation in susceptibility to *M.tb* infection and progression to active disease has been observed in different ethnic and geographic populations ^76, 77^. A study of >25,000 residents in racially-integrated nursing homes in Arkansas, USA, found that 13.8% of African-American compared with 7.2% of Caucasian residents had evidence of a new *M.tb* infection ^78^. Furthermore, in a study of three TB outbreaks in two prisons, African-Americans had approximately twice the relative risk compared with Caucasians of becoming infected with *M.tb* ^78^. However, although these studies largely controlled for environmental factors, confounders such as differing vitamin D levels cannot be ruled out.

More recently, genotyping technologies have supported a role for genetic ancestry in TB susceptibility. A case-control study genotyped a panel of ancestry informative markers to estimate the ancestry proportions in a South African Coloured [sic] population. African ancestry (particularly San ancestry) was higher in TB cases than controls, and European and Asian ancestries were lower in TB cases than controls ^76^. However, a limitation is that the study did not adjust for socioeconomic confounders. Differences in alleles that encode components of the immune response provide a possible mechanism for ethnic variation in TB susceptibility. Indeed, a study of African and Eurasian pulmonary TB patients in London indicated ethnic differences in the host inflammatory profile at presentation including lower neutrophil counts, lower serum concentrations of CCL2, CCL11 and DBP, and higher serum concentrations of CCL5 in those of African ancestry ^79^. These differences became more marked following initiation of antimicrobial therapy, and were associated with ethnic variation in host genotype but not *M.tb* strain ^79^.

Attributing variation in TB incidence between populations to genetic differences has been criticised, particularly due to fears that a discussion of ‘innate’ differences will perpetuate the naturalisation of inequalities in TB incidence ^80^. Moreover, ‘race’ is a problematic concept, acting only as a proxy for genetic variation that is continuously distributed according to ancestry ^81^. However, to recognise that there is some genetic variation in TB susceptibility that broadly aligns with ethnic groups is not to deny that there are other factors contributing to differences in TB incidence, as discussed in this review.

Host-pathogen co-evolution is a likely driver of variation in TB susceptibility in different human populations ^82^. *M.tb* has been co-evolving with humans for millennia, with evidence that humans were exposed before the Neolithic transition ^83^. The differential susceptibility of particular populations may be based on *M.tb* exposure history, with long-term exposure resulting in strong positive selection for resistance-related alleles. There is evidence from European colonialism that previously underexposed populations are more susceptible to TB, which played a large part in the deaths of many Qu’Appelle Indian and Inuit populations in Canada ^84, 85^. Similarly, in contrast to Europeans, Southern African populations have only been exposed to modern *M.tb* strains relatively recently ^76^. It has been suggested that selection pressure for resistance would have been strongest in areas of high population density. Accordingly, duration of urban settlement is correlated with the frequency of the SLC11A1 1729 + 55del4 allele which plays a role in natural resistance to intracellular pathogens including *M.tb* ^86^. Lower rates of TB among those of European ancestry could be due to centuries of exposure in densely populated settlements driving the evolution of increased resistance.

## Vitamin D deficiency

It has long been recognised that low vitamin D levels are associated with active TB, with sunlight exposure in sanatoria and direct administration of vitamin D commonly used as treatments prior to the advent of antibiotics ^87^. Today, evidence supporting a link between TB and vitamin D deficiency is accumulating, although the association is complex ^88– 91^. A meta-analysis indicated a 70% probability that, when chosen at random from a population, an individual with TB disease would have a lower serum vitamin D level than a healthy individual ^92^, although the direction of causality is not clear. It has been demonstrated that 1,25(OH) _2_D, the active metabolite of vitamin D, promotes the ability of macrophages to phagocytose *M.tb* and enhances the production of cathelicidin LL-37, an antimicrobial peptide that has direct bactericidal activity and attracts other immune cells to the site of infection ^93^. There is evidence that a drop in serum vitamin D compromises the immune response and can lead to reactivation of LTBI ^94^. Vitamin D deficiency may also be a risk factor for extrapulmonary dissemination of TB, which is more common among migrants and those of non-white ethnic groups ^95, 96^.

Vitamin D can be acquired from the diet or endogenously synthesised in the skin by the photolytic action of solar UV light on the precursor molecule 7-dehydrocholesterol ^97, 98^. Certain migrant and ethnic minority groups are at a greater risk of vitamin D deficiency ^99– 101^. Indeed, vitamin D levels have been shown to be lower among Asian children living in England compared with children of the same age in the general population ^102^. Vegetarians are at increased risk of vitamin D deficiency since oily fish are a major dietary source ^103^, and Hindu Asians are more frequently vegetarian than the general population due to socio-religious factors ^104^. A study of Asian immigrants with TB disease in Wandsworth found that Hindus were at higher risk of contracting TB than Muslims ^105^. Darker skin pigmentation also increases risk of deficiency, as melanin reduces the efficiency of vitamin D synthesis from UVR ^101, 106^. Hindu women in particular have been found to be at high risk, as in a 1976 study by Hunt *et al*. they spent on average only 2.5 hours a week outside for cultural reasons, whereas men were exposed to sunlight travelling to work ^107^.

1,25(OH) _2_D mediates its immune activity through binding to the vitamin D receptor (VDR) on target cells; thus receptor abnormalities as well as vitamin D deficiencies may impair host immunity to *M.tb* ^108^. Some polymorphisms in the *VDR* gene increase susceptibility to TB, while others increase resistance ^109, 110^. In a systematic review of seven studies comparing the prevalence of *VDR* polymorphisms in TB patients and healthy controls, BsmI and FokI *VDR* polymorphisms were found to increase TB susceptibility ^111^. The *VDR* gene shows striking genetic variation in allele frequency between populations ^112^. Moreover, certain polymorphisms play different roles in different populations ^111^, although further research is required to elucidate how this translates into variation in patterns of susceptibility and resistance to TB in different ethnic groups. Epigenetic variation in the *VDR* gene in different ethnic groups, arising from differential exposure to environmental factors, may influence gene regulation and therefore contribute to differential TB susceptibility. Indeed, methylation variable positions at the 3' end of *VDR* have been identified that are significantly correlated with ethnicity and TB status ^113^.

## Co-morbidities

Risk of progression to active TB disease is increased in those with conditions that impair immunity, such as diabetes mellitus (DM), human immunodeficiency virus (HIV) and chronic kidney disease (CKD) ^114^. Certain migrant and ethnic groups are at a higher risk of these conditions; diabetes mellitus disproportionately affects South Asians whereas HIV is more prevalent among those of African origin, and chronic kidney disease affects both groups.

a) Diabetes mellitus

Clinicians have noted a possible association between TB and DM since the early 20 ^th^ century ^115^. More recently, a meta-analysis of 13 cohort studies found that DM increases the risk of active TB 3.11-fold ^116^. A causal relationship between DM and impaired immunity to TB is supported by studies of diabetic mice, which have higher bacterial loads when infected with *M.tb* than non-diabetic mice ^117^. DM-TB comorbidity increases both the risk of new and reactivational TB ^118^, although diabetes as an independent risk factor was associated with only a modest overall increased risk of TB in a UK General Practice cohort ^119^. Various mechanisms have been suggested including impaired immune function due to DM, complications of DM, and deficiencies in vitamins A, C and D associated with both TB and DM risk ^120^.

Type 2 DM (T2DM) and associated risk factors, especially obesity, show marked associations with ethnicity ^121^. In the UK, obesity and T2DM risk is significantly higher among South Asians (including those of ISC origin), and moderately higher among black African-Caribbeans compared with white Europeans ^122^. The prevalence of DM among South Asians in England was 14% in 2010; approximately double the 6.9% prevalence in the general population ^123^. Some studies have suggested that ethnic differences in T2DM can be explained by differences in socio-economic status ^124^, while others do not support this ^121^. It is clear there are complex genetic and environmental explanations for ethnic differences in T2DM prevalence, which are beyond the scope of this review (for example, see 125).

b) Human immunodeficiency virus

Infection with HIV is the strongest known risk factor for the development of TB disease ^126^. TB-HIV co-infection synergistically worsens both conditions, leading it to be termed ‘the cursed duet’ ^127^. HIV increases both the risk of rapid progression to active disease following infection and reactivation of LTBI, with an increased risk of TB throughout the course of HIV-1 disease ^128, 129^ and incidence rate ratios >5 when averaged across all levels of immunodeficiency ^130^. The depletion of CD4+ T cells associated with HIV-1 infection is thought to play a major role in the increased risk of TB and its extra-pulmonary dissemination in infected individuals, as *M.tb* infected macrophages require CD4+ T cells to augment intracellular clearance ^131^. Furthermore, peripheral blood lymphocytes of HIV-positive patients produce less interferon-γ when exposed to *M.tb in vitro* than those of HIV-negative patients ^132^. These and other possible immune mechanisms such as chronic inflammation promoting an immunoregulatory phenotype and attenuation of phagocytosis have been recently reviewed ^133^.

A systematic review on the prevalence, incidence and mortality of HIV-TB co-infection in Europe observed a disproportionate vulnerability of migrants to co-infection across studies ^134^. Given that only 3.8% of TB cases in England in 2015 involved co-infection with HIV ^14^, TB-HIV co-infection cannot be considered a major driver of higher TB incidence among migrants and ethnic minorities in the UK; although this group represents an important target in the prevention of *M.tb* infection and progression to active disease. TB-HIV coinfection undoubtedly plays a role in explaining the higher TB incidence rates among those of African origin. In 2015, 81.8% of TB-HIV co-infected cases in England were foreign-born, of which 68.7% were born in sub-Saharan Africa ^14^ This reflects the global distribution of TB-HIV co-infection. In the WHO African region, 38% of new TB cases were co-infected ^130^. In turn, the global pattern of TB-HIV co-infection reflects the global distribution of HIV: 69.5% of all people living with HIV are in the WHO African region ^135^.

c) Chronic kidney disease

The association between CKD and TB was first reported in 1974 ^136^, and has been subsequently confirmed by several studies ^137– 139^. The mechanism is thought to be impaired immunity: CKD is associated with functional abnormalities in various immune cells, such as B and T cells, monocytes, neutrophils, and natural killer cells ^140^. This increases the risk of both newly acquired and reactivated TB. Furthermore, immunosuppressive medications in kidney transplant patients are aimed at T cell-mediated immunity, which is central to maintaining TB latency in LTBI individuals ^141^. Patients with CKD are 10–25 times more likely to develop active TB ^142^.

Ethnic minorities in the UK are at a 3–5 times higher risk of developing CKD ^143^. A study in London from 1994–1997 found that the incidence rate among white Caucasians was 58/million adult population per year, 221 among South Asians, and 163 among African-Caribbeans ^144^. More recently, in a study of CKD patients with TB in South East London, 74% were born outside of the UK ^145^. CKD also interacts with other risk factors that contribute to higher incidence of TB among migrants and ethnic minorities. DM patients are 4–5 times more likely to have CKD ^146^, CKD patients are more likely to have low vitamin D levels ^147^, and CKD is a complication associated with HIV ^148^. Furthermore, CKD disproportionately affects economically disadvantaged groups, possibly due to the direct impact of poverty or malnutrition, or indirect effects of poverty-associated co-morbidities including DM and HIV ^149^. Given the complex interactions between multiple risk factors, it is difficult to establish the direction of causality.

## Socio-economic status

The association between deprivation and TB has long been recognised, leading it to be dubbed a “social disease” ^150^ and “poverty’s penalty” ^151^. There is a strong socio-economic gradient in TB burden between and within countries and communities, with economically disadvantaged groups having the highest risk ^152^. The importance of social factors in TB risk is supported by McKeown’s observation that a considerable proportion of the decline in TB-associated mortality occurred before the advent of antibiotics and the BCG vaccine, implicating improved living standards and nutrition as the main drivers ^153^. Szreter contests the McKeown thesis, emphasising the key role of public health measures in regulating the urban environment ^154^. Either way, it is clear that TB disproportionately affects the socially and economically marginalised, with a recognised role for poverty, homelessness, and overcrowding in both the spread of infection and number of active cases ^18^. In the UK in 2009, TB cases among the homeless were 20 times higher than the general population at 300 cases per 100,000 ^155^. In a study of London districts, the TB notification rate increased by 12% for every 1% rise in the number of people living in overcrowded conditions ^156^.

The wider social determinants of health are entwined with ethnicity, meaning that ethnic socio-economic disparities throughout the life course often lead to health inequalities ^157^. There are marked economic inequalities between ethnic groups in the UK, with both Asian and black ethnic groups having lower employment probability than the population average ^158^. Alongside economic issues of unemployment, low income and poor working conditions, migrants and ethnic minorities are also more likely to face problems of homelessness, poor housing, and overcrowding ^159^. Foreign nationals accounted for 13% of the general UK population in 2015 ^160^, but 20% of the homeless population ^161^. In 2011, dwellings with a Household Reference Person (HRP) from a minority ethnic group represented 16.1% of all households in England and Wales, but 47.9% of overcrowded households. The most commonly overcrowded households were those with a Bangladeshi HRP (30.2% overcrowded), followed by Pakistani (22.3%) and black-African (21.8%) ^162^.

King rejects what he terms ‘essentialist’ explanations for the higher TB incidence of migrants and ethnic minorities, which claim that TB disproportionately affects certain groups due to intrinsic differences, which may be biological, genetic, physiological or cultural. Instead he suggests that “Disparities in health that may at first seem to arise from essential racial or ethnic differences are often in fact the result of contingent socioeconomic differences” ^18^. Similarly, Farmer rejects psychological or cultural explanations, emphasising that “tuberculosis is inextricably tied to poverty and inequality”. He criticises studies that neglect to address the political-economic forces that shape TB distribution, and calls for anthropologists to pay more attention to structural violence (the systematic ways in which social structures disadvantage individuals) and social inequality ^163^.

King and Farmer claim that, given that socio-economic status affects TB risk, biological and genetic approaches are largely irrelevant ^18, 163^. Similarly, the medical anthropologist Singer criticised the use of adaptation as a conceptual tool on the basis that such explanations ignore how the political economy shapes the environment that humans adapt to. She argues that differential mortality between socio-economic groups is “unnaturally selected” by the conditions created to further the interests of the dominant class ^164^. However, as discussed, there is evidence to suggest that adaptation resulting from host-pathogen co-evolution influences direct and indirect genetic susceptibility to TB infection and progression to active disease. Perhaps as Mason *et al*. suggest, a more constructive approach is required, recognising that “the social model is an important complement to the biomedical model” ^165^.

Given the complex association between ethnicity and socio-economic status, it is hard to disentangle the extent to which socio-economic disadvantage influences TB incidence in migrant and ethnic minority populations ^166^. One study in children from Leeds found that overall, ethnicity explained a high proportion of TB incidence independently of deprivation and population density, although for non-South Asian children, the strongest risk factor was deprivation ^167^. Similarly, a study in Liverpool suggested an association between ethnicity and TB incidence that was independent of deprivation level ^168^, and a study investigating TB trends in England in 1999–2003 indicated that affluent ethnic minority groups are still at greater risk ^166^. It has been suggested that the absence of a strong correlation between deprivation and *M.tb* infection in the South Asian community may be due to the smaller relative differences in deprivation within this group than across the general population ^169^. Indeed, a study in Newham found an association between the proportion of non-white residents and TB diagnosis in each ward, but no association with deprivation as the borough as a whole was deprived ^170^.

There is significant heterogeneity in the role that social risk factors play in increasing TB risk in different migrant and ethnic groups. Among UK-born cases notified in 2010–2016, 33.1 % of those in the black-Caribbean ethnic group had at least one social risk factor (homelessness, imprisonment, drug or alcohol misuse), higher than any other ethnic group ^14^. 19.4 % of black-Caribbean cases were drug users, and 18.6% had a history of imprisonment. The countries of origin with the highest number of homeless TB cases were Somalia, at 95 cases, and Eritrea, at 91 cases ^14^. This suggests that socio-economic disadvantage may play a particularly important role in explaining higher TB incidence among the black-African and black-Caribbean ethnic groups.

## Experiences of migration

The difficulties faced during and shortly after migration may increase risk of progression to active disease by compromising immunity, including poor nutrition, concurrent poor health, socioeconomic marginalisation, and the stress of relocation ^18^. In an anthropological study of illegal Chinese immigrants with TB in New York, it was found that migrants often experience shortages of food and water during long migratory journeys. Upon arrival, temporary residence in detention centres or illegal refuges is associated with overcrowding and malnutrition ^171^. Migrants then face additional challenges including loss of a social support network, communication issues, discrimination, and acculturation ^172^. Ho calls for a focus on the macro-level structural forces that shape TB risk on migratory journeys, such as a lack of government regulation and exploitation by human traffickers ^171^.

Psychological effects include higher rates of anxiety among refugees and asylum seekers compared with the general population or other migrant groups ^173^, and poorer mental health in forced compared with voluntary migrants ^174^. Furthermore, Africans in Britain are at a higher risk of mental illness than non-Africans ^175^, and survey data suggests that immigration is a primary cause of mental distress in about 40% of Africans in the UK ^176^. It has been suggested that the psychological stress and depression associated with migration may play a role in increasing risk of progression to active disease, potentially via neuroendocrine pathways or a negative effect on the cell-mediated immune system ^177^.

Importantly, rather than transporting active cases of TB across national borders, the majority of immigrant cases of active TB disease develop following arrival in the UK ^20^. This supports King’s assertion that “The higher rate of TB among immigrants owes as much to the hardships they face during and shortly after migration, as it does to their country of origin” ^18^. Further research is required to establish the extent to which stress and adverse migratory journeys affect specific migrant groups. However, experiences of migration are likely to contribute at least in part to the higher rates of active TB among migrants compared with UK-born ethnic minorities and the general population, especially for those who are marginalised or are travelling illegally.

## Treatment-seeking

Knowledge about TB among migrants and ethnic minorities is shaped by cultural beliefs, often arising from experiences in the country of origin ^178^. Certain ideas, including misconceptions about TB causation, transmission and risk, can act as barriers to clinical treatment. Gerrish *et al*. suggest that “TB is not just a medical disease to be treated with antibiotic therapy but an entity with historical and cultural roots” ^179^. Several studies have identified widespread misconceptions about TB causation and transmission among migrant communities, and a limited understanding of LTBI in particular ^172^. The disease has been variously attributed to climate conditions ^180^, poisoning, pneumonia ^181^, exposure to chemical products ^182^, and witchcraft ^183^. Several members of a focus group of Somali women believed TB to be a punishment for past ill deeds ^178^.

Migrants may feel a false sense of having ‘left behind’ the high risk of TB in their country of origin ^172^, and TB may be considered by migrants to be a different, more severe, disease in their country of origin ^184^. In some cases, TB may be thought of as incurable due to poor health services in low-income countries ^185^. Moreover, immigrants may favour traditional systems of care and healing over Western medicine upon arrival ^186^, making them more likely to turn to traditional folk healers, self-diagnosis or self-medication before accessing public healthcare facilities ^159^. Cultural beliefs that lead to delays in treatment-seeking and reduced adherence to treatment may increase the risk of TB transmission within such communities.

Conversely, some have suggested that the cultural beliefs held by migrants are not barriers to treatment-seeking, but rather promote such behaviour. The higher prevalence of TB in migrants’ countries of origin could lead to greater awareness; as Bakhshi argues, “people born in developing countries are too familiar with the disease to neglect it” ^29^. Moreover, Ho describes how traditional Chinese medical beliefs are often complementary to clinical TB treatment in New York, such as through the use of traditional Chinese medicine to reduce the side effects of anti-TB drugs ^171^.

TB-related stigmatisation of immigrants has been reported in multiple studies (reviewed in 172). Stigma is defined as “the situation of the individual who is disqualified from full social acceptance”, and is therefore “reduced in our minds from a whole and usual person to a tainted, discounted one” ^187^. Some cultures consider TB to be sinful and dirty ^178^. The feelings of guilt and shame ^188^ and risk of rejection and discrimination ^189^ that may result from stigmatisation affect attitudes towards diagnosis, treatment and prevention, and therefore hinder control of TB and facilitate its transmission within certain migrant and ethnic groups ^190^. Sufferers may hide their illness to avoid stigma and discrimination and protect personal or family dignity ^183^. One study indicated that stigma prevented some immigrants from sharing information with their doctors, even TB-related symptoms ^191^. Furthermore, patients may be less likely to identify contacts due to concerns about social repercussions, meaning that subsequent preventable TB cases may occur ^179^. Feelings of stigma produced by attitudes in the country of origin are likely to be exacerbated by the negative stereotyping of migrant groups as ‘dirty’ or ‘diseased’ due to the association of TB with immigrants, which may lead to xenophobia and discrimination of sufferers ^185^, termed ‘sociomedical racism’ by McBride (1991) ^192^.

The Somali community in the UK provides an informative case study of the socio-cultural meaning and perceived consequences of TB. In Somalia, TB is associated with extreme stigma and social isolation. In a focused ethnography of Somali-born UK residents, Gerrish *et al*. found that interviewees tended to base their attitudes towards TB on those prevalent in Somalia ^179^. The stigma associated with TB led to expectations of social isolation, shame and loss of self-worth, sometimes extending to the whole family. Although most had an understanding that TB is contagious, it was also commonly believed that people remain infectious after treatment, as TB was often thought to be hereditary and therefore impossible to eradicate. This led to fears that friends would not resume normal social interactions after treatment, and that a diagnosis would jeopardise marriage prospects. Therefore, sufferers tended to isolate themselves or conceal their illness. In reality, anticipated consequences tended to be worse than actual experiences of discrimination, but felt stigma was nonetheless a powerful deterrent to disclosing illness, leading to delays in diagnosis and treatment ^179^.

## Access to healthcare

Migrants may have difficulties establishing ‘entitlement’ to good healthcare ^193^. For example, a study in the UK found that only 32.5% of new migrants who were instructed to register with a GP had done so, and the migrant groups with the smallest proportion registered were likely to have greatest need ^194^. This is consistent with the Inverse Care Law, that those with the greatest need are least able to access healthcare services ^195^. Various studies have found that migrants face barriers in accessing healthcare services for TB diagnosis or treatment. These include lack of awareness of the local health system, including availability of free services ^196^, language barriers ^197^, and fears about loss of privacy due to the use of interpreters ^184^. Therefore, even in cases where there are minimal geographic or economic barriers to accessing health facilities, there are often racial, linguistic and cultural barriers to using these facilities effectively and adhering to treatment regimens ^198^. Worryingly, institutional racism within the NHS may pose a further barrier to healthcare access for both migrants and ethnic minorities. There is evidence that, when commissioning services, Trusts often fail to meet the needs of black and ethnic minority populations, which may reflect a lack of diversity among the NHS senior workforce ^199– 201^.

Studies have found that migrants face various structural barriers to accessing healthcare services, such as transport difficulties associated with poor services in deprived areas ^178^, and rigid opening hours for medication that do not fit with the working hours and lifestyles of patients ^202^. Moreover, economic barriers include not only direct costs associated with illness, such as the costs of repeated journeys to clinics for treatment, but also indirect costs including losing a job or being evicted by a landlord ^203^. Farmer criticises anthropological investigations for conflating structural violence with cultural difference, tending to exaggerate the role of patient agency and minimise the role of poverty and the barriers that it creates to accessing adequate care and completing treatment ^163^. Nevertheless, whether structural or cultural, barriers to healthcare access among migrant and ethnic groups can lead to delays in diagnosis and treatment, resulting in increased transmission and incidence.

Access to healthcare services varies across different migrant populations. Although treatment of TB is free for all in the UK, refugees and asylum seekers have poorer access to health services ^204^. Irregular residence status is likely to lead to significant delays in seeking medical assistance, due to uncertainties surrounding entitlement to services and fears of deportation, since TB patients can be legally deported while receiving ongoing treatment ^205^. Furthermore, irregular migrants may face difficulties in completing long-term TB treatment, which involves repeated consultations, if they do not have housing or employment and are short-term residents. Irregular migrants are also less likely to be willing to provide details of their migratory route ^184^ and provide information about contacts ^183^. Contact tracing is further compromised given the high mobility of migrants, and the fact that many do not reside at their official address, but with family and friends ^184^.

Conversely, there is evidence to suggest that UK-born cases experience longer delays from symptom onset to commencement of treatment than foreign-born cases ^15^. Moreover, TB treatment completion is actually marginally higher in migrants (85%) than the UK-born (81%) ^50^. However, these observations are problematic in that UK-born TB cases are often drawn from homeless individuals, problem drug users and prisoners, and so are frequently lost to follow-up and poorly adherent ^206^; they are not representative of the UK-born population as a whole. Furthermore, migrants are at a higher risk of contracting TB due to the various unique factors discussed (including genetics, vitamin D deficiency, co-morbidities, and experiences of migration), which do not apply to the UK-born, making socio-economic issues the key driver of TB incidence in this group. Indeed, in 2016, 2.4 times as many UK-born cases (20%) as foreign-born cases (8.2%) had at least one social risk factor (drug misuse, alcohol misuse, homelessness, or imprisonment) ^14^, which is incongruent with the higher overall rates of deprivation in foreign-born compared with UK-born populations ^32^.

## Conclusions

It is a common misconception that migrants have a higher incidence of TB disease compared with the general population simply because they ‘import’ it from abroad. Bakhshi suggests that they “present a tuberculosis picture from the country of origin and not the United Kingdom where the disease eventually manifests” ^29^. Indeed, differential pathogen exposure can explain much of the higher incidence of TB among migrants and ethnic minorities, due to both pre-migration residence in high-incidence countries and maintenance of transnational links with the country of birth or ethnic origin. However, positing this as the sole driver fails to address the complex interplay of factors driving the vulnerability of particular migrant and ethnic groups to infection and progression to active disease. These include genetic susceptibility, vitamin D deficiency due to climatic and dietary factors, co-morbidities including DM, HIV and CKD, socio-economic deprivation, and factors linked to the experience of migration itself. Furthermore, certain migrant and ethnic groups face barriers to accessing treatment including cultural differences in treatment-seeking behaviours, stigmatisation of sufferers, and barriers to healthcare access. As stated by Offer *et al*., “TB in ethnic minorities does not occur in isolation but against a backdrop of socioeconomic, political and cultural context that affects their knowledge, attitudes and behaviours” ^169^. The resultant delays in diagnosis and treatment lead to increased transmission and incidence in these communities.

In this way, there are factors disadvantaging migrants and ethnic minorities at each stage of the disease, relating to risk of pathogen exposure, vulnerability to infection, development of active disease, and access to treatment. Although heterogeneity between and within broad migrant and ethnic groups leads to variation in risk at each of these stages, there is a net effect of higher incidence among migrants and ethnic minorities compared with the general UK population. It is important to understand the complex and multifactorial drivers of this disparity in order to implement effective policies for tackling TB in these vulnerable groups. Currently, migrants from countries with high TB incidences are screened for active TB before entry to the UK. However, to complement such measures, which only consider the driver of differential pathogen exposure, more consideration is needed regarding policies that address the factors making migrants and ethnic minorities more vulnerable to reactivation of LTBI following their arrival in the UK. This might include vitamin D supplementation, measures targeting co-morbidities, and policies that promote socio-economic equity and migrant rights. In order to reduce delays in diagnosis and treatment, and thereby minimise transmission within migrant and ethnic minority communities, increased health education on TB causation, risk and transmission is required, as well as tackling stigmatisation of vulnerable groups. It is also important to raise awareness of migrants’ entitlement to diagnosis and treatment through the NHS, alongside reducing cultural and economic barriers to its access.

## Data availability

The data referenced by this article are under copyright with the following copyright statement: Copyright: © 2018 Hayward S et al.

Data associated with the article are available under the terms of the Creative Commons Zero "No rights reserved" data waiver (CC0 1.0 Public domain dedication). 

No data are associated with this article.
