# Supplementary material for: Factors influencing the higher incidence of tuberculosis among migrants and ethnic minorities in the UK
Source: F1000Res. 2018 Aug 20;7:461. Originally published 2018 Apr 13. [Version 2] doi: 10.12688/f1000research.14476.2 (PMC6107974; doi:10.12688/f1000research.14476.2)
Supplement: Peer review history

# Peer review: Referee response for version 2

**DOI:** 10.5256/f1000research.17399.r37375

**Published:** 2018-08-21

**Authors:** Manish Pareek

**Affiliations:** 1 Department of Infection, Immunity and Inflammation, University of Leicester, Leicester, UK

The authors have made all required changes. No further comments.

I have read this submission. I believe that I have an appropriate level of expertise to confirm that it is of an acceptable scientific standard.

# Peer review: Referee response for version 1

**DOI:** 10.5256/f1000research.15761.r34098

**Published:** 2018-07-09

**Authors:** Manish Pareek

**Affiliations:** 1 Department of Infection, Immunity and Inflammation, University of Leicester, Leicester, UK

Thank you for asking me to review this manuscript which reviews the factors influencing the higher incidence of TB in migrants and ethnic minorities.

 Introduction

- The authors should clarify that the symptoms of active disease both site-specific and systemic and list site-specific symptoms.

Epidemiology

- Although a mainly UK based paper, is there evidence from other high-income countries relating to transmission in migrant communities?

Differential exposure

- Whilst I agree travel is potentially important as a risk factor for acquiring TB infection, the evidence at present is weak. I think the authors need to nuance this section and clarify that the data is weak and further work is required. In particular type of travel is important. Migrants working in healthcare overseas as volunteers etc will be at higher risk.

Vitamin D

- The impact of vitamin D is difficult to tease out and I think the authors should cite the work of Pareek and colleagues which suggests that vitamin D levels (i.e. deficiency) are associated with EPTB.

Impact of strain

- I would like more discussion on the host-pathogen interaction and impact of strain type on TB phenotype. See Pareek et al's work in this area.

Diabetes

- The relative risk of TB in DM patients seems lower in the UK. Have they seen the work from David Moore's group on the CPRD cohort?

HIV

- Whilst I agree that the absolute proportion of TB-HIV cases is low it should be borne in mind that incidence in this group is relatively high and they are important group to prevent TB infection and progression in. 

First generation vs. second generation

- I would have liked some discussion in the manuscript relating to why second generation individuals (i.e. UK born but with parents born overseas) are at increased risk of TB. This is often neglected but needs to be evaluated.

I have read this submission. I believe that I have an appropriate level of expertise to confirm that it is of an acceptable scientific standard, however I have significant reservations, as outlined above.

# Peer review: Referee response for version 1

**DOI:** 10.5256/f1000research.15761.r33177

**Published:** 2018-04-27

**Authors:** Jessica L. Potter

**Affiliations:** 1 Blizard Institute, Queen Mary University of London, London, UK

This is an important review and good addition to the literature in that it combines both the biomedical and the social determinants of TB risk amongst populations that experience a disproportionate burden of disease in the UK. Great concluding section in particular. "Given the complex association between ethnicity and socio-economic status, it is hard to disentangle the extent to which socio-economic disadvantage influences TB incidence in migrant and ethnic minority populations" - I suggest this needs to be more explicit from the start and particularly as you risk, perhaps (my reading of it) conflating the migrant and ethnic-minority experience thus contributing to an idea that non-white = non British.

 I note the reference to Nancy Krieger's work on the embodiment of risk across a life course. Considering TB through this ecosocial perspective allows an analysis of the interplay between the biomedical and the social. I wonder whether the article would flow better and provide a more critical reading if situated within this framework and, in particular, framing the literature from a 'distal' or macro (social determinants) perspective down to more proximal factors might provide a better reading. In terms of considerations of factors that contribute to risk I think that placing discussions of genetic susceptability at the start somewhat undermines the well founded concerns highlighted in the social determinants section - that our understanding of TB and research investment heavily focuses on the biomedical rather than the social.

 The section about genetic susceptibility is perhaps a little uncritical and positions the association between ethnicity and TB risk at a genetic level as taken for granted. For a critical reading of Cummins research in relation to race and racism, for example, chapters 1 and 2 (the rise and fall of race) in Discovering TB by Christian McMillen.  

 I also note you use the term 'colored' to describe participants -  I realise this is in the title of that study. As this term has racist origins I would suggest either adding [sic!] after it or altering the term to more accurately describe the population the research was conducted amongst (the former is probably easiest!). 

 A few other points:

 - 'Hindu-Asians are more frequently vegetarian' - than who?

 - 'Globalisation and capital flows' are drivers of migration - the reference is a report on migration and cities...is there a better reference?

 - 2015 PHE data provided - can this be updated? 

 - The section on health-seeking states "The disease has been variously erroneously attributed to climate conditions...." - Please can you delete 'erroneously': You previously discuss vitamin D and migration - both related to 'climate conditions' as possible risk factors for TB (I realise this is not necessarily what the participants were getting at in their interviews but still think best to remove it) My other reason for removing it is that it undermines what is ''truth' as experienced by patients.  

 - 'Non-compliance' - I tend to use adherance as per NICE guidelines https://www.nice.org.uk/guidance/cg76

 - "Farmer criticises anthropological investigations for conflating structural violence with cultural difference" - The report the 'snowy white peaks of the NHS' and the McPherson report in the wake of the Stephen Lawrence inquiry talked about institutional racism within the NHS and I wonder whether this would be useful to mention as you talk about cultural barriers in relation to language, knowledge of health systems etc but, although you mention 'racial' barriers it is within the context of talking about migrants, rather than British-born BAME communities whose challenges in accessing care you don't specifically address.

I have read this submission. I believe that I have an appropriate level of expertise to confirm that it is of an acceptable scientific standard.
